# Ionic Liquids as Protein Crystallization Additives

**DOI:** 10.3390/cryst11101166

**Published:** 2021-09-24

**Authors:** Crissy L. Tarver, Qunying Yuan, Marc L. Pusey

**Affiliations:** 1 Department of Structural Biology, Stanford University School of Medicine, Stanford, CA 94305, USA; 2 Department of Biological and Environmental Science, Alabama A&M University, Normal, AL 35762, USA; 3 Department of Chemistry, University of Alabama in Huntsville, Huntsville, AL 35805, USA

**Keywords:** ionic liquids, Hofmeister series, crystallization, crystallization additives, inorganic pyrophosphatases

## Abstract

Among its attributes, the mythical philosopher’s stone is supposedly capable of turning base metals to gold or silver. In an analogous fashion, we are finding that protein crystallization optimization using ionic liquids (ILs) often results in the conversion of base protein precipitate to crystals. Recombinant inorganic pyrophosphatases (8 of the 11 proteins) from pathogenic bacteria as well as several other proteins were tested for optimization by 23 ILs, plus a dH_2_O control, at IL concentrations of 0.1, 0.2, and 0.4 M. The ILs were used as additives, and all proteins were crystallized in the presence of at least one IL. For 9 of the 11 proteins, precipitation conditions were converted to crystals with at least one IL. The ILs could be ranked in order of effectiveness, and it was found that ~83% of the precipitation-derived crystallization conditions could be obtained with a suite of just eight ILs, with the top two ILs accounting for ~50% of the hits. Structural trends were found in the effectiveness of the ILs, with shorter-alkyl-chain ILs being more effective. The two top ILs, accounting for ~50% of the unique crystallization results, were choline dihydrogen phosphate and 1-butyl-3-methylimidazolium tetrafluoroborate. Curiously, however, a butyl group was present on the cation of four of the top eight ILs.

## 1. Introduction

Room temperature ionic liquids (RTILs), generally defined as salts that are liquid at ≤100 °C, started attracting increased attention at the turn of the century. Although they were known well before, it was not until this time that the advantages and utility of their unique properties became apparent. Among these properties are low vapor pressure, typically high viscosity, and high thermal stability. Because of the enormous potential number of structures, lending almost exquisite tuning of the properties of RTILs, they have also become known as designer solvents.

The first application of an IL for protein crystallization was the use of ethyl ammonium nitrate (EAN) for the crystallization of chicken egg white lysozyme [1] (Garlitz et al., 1999). Since then, several studies into the crystallization of proteins with ILs have been carried out. Subsequent crystallization experiments using four model proteins with three ILs, two based on the 1-butyl-3-methylimidazolium and one on the 1-butyl-1-methylpyrollidinium cation, each with a different anion, were carried out [2]. A broader survey, involving 16 different ILs on the crystallization of 6 model proteins, was conducted [3]. The IL parent cations tested included imidazolium, phosphonium, ammonium, and pyridinium, with a variety of anions. Protic ILs were tested as protein crystallization additives [4]. Ten ILs with cations based on the ethyl ammonium moiety were tested. This study was designed to test the effects of different anions. For all of the above IL-protein crystallization studies, the ILs were found to be best employed as additives, often effective in improving the size, (visual) quality, and reproducibility of the crystallization process. As the effective ILs were different for each protein, no conclusions about systematic effects due to IL structure could be derived.

ILs have been found to improve the bulk crystallization of proteins in stirred reactors [5]. The IL C_4_[mim]-BF_4_ has been reported for use as the precipitant in the crystallization of the common model proteins lysozyme and thaumatin [6]. ILs are also finding application in the stabilizing of protein structure. Lysozyme was thermally stabilized by ethanolammonium formate [7], which also gave near-complete renaturation upon cooling. ILs have been shown to be effective solvents for protein refolding [8,9]. Thermal stability has been shown to be a major factor in protein crystallization [10,11]. ILs, used as additives, have been shown to promote the thermal stability of lysozyme crystals [12]. The ability of ILs to increase lysozyme solubility with temperature has also been shown [13]. Not surprisingly, IL structure has been shown to also affect protein stability [14].

To date, only cursory surveys have been carried out to identify which, if any, IL structural features (of the cation or anion) are most useful for protein crystallization, perhaps not surprising given the enormous potential range of structures. This work reports on a starting investigation of what IL structural features most optimize them for macromolecule crystallization purposes. Herein, we have used the crystallization of a range of inorganic pyrophosphatases (IPPases) from pathogenic bacteria, plus three other proteins, to test the use of ILs as additives. The primary experimental goals were to comparatively determine the effects of IL structure on protein crystallization and to determine if ILs could be used to systematically improve less desirable crystallization trial outcomes (urchins, needles, dendrites, spheroids, precipitate, etc.) to more useful outcomes such as 3D crystals.

The bulk of the proteins used in these studies are recombinant inorganic pyrophosphatases derived from pathogenic microbes. Soluble IPPases are divided into three families—Family I, which are magnesium dependent [15,16]; Family II, which are manganese dependent [17,18]; and Family III, which have only recently been described. This IPPase family shows a Ni++ dependence and belongs to the haloacid dehalogenase superfamily of proteins [19]. Family I IPPases are single-domain proteins that form homohexamers, dimers of trimers, in prokaryotes and homodimers in eukaryotes. Family II IPPases are typically homodimers.

## 2. Materials and Methods

### 2.1. Materials

Proteins: Human apo transferrin was from Sigma (Tuscaloosa, AL, USA, Cat.# T-1147) and used without further purification. The proteins Tt189 (nucleoside diphosphate kinase) and RrP42 (archaeal exosome complex protein) from Thermococcus thioreducens were cloned, expressed, and purified as previously described [20]. Nucleic acid sequences corresponding to the inorganic pyrophosphatases (IPPases) from Haemophilus influenzae (Hi), Klebsiella pneumonia (Kp), Acinitobacter baumannii (Ab), Campylobacter jejuni (Cj), Salmonella typhi (St), Francisella tularensis (Ft), Streptococcus pneumonia (Spn), and Streptococcus pyrogenes (Spy) were synthesized using previously described methods [21]. The synthesized genes were inserted between NdeI and BamHI sites of pET3a (Novagen, Madison, WI, USA) through homologous recombination in vivo. To facilitate protein purification, a His6-tag (MHHHHHHQ) was added to the N-terminus of the proteins. The plasmids were propagated in *E. coli* strain DH5*α* (Genlantis, Inc. San Diego, CA, USA). Error-free clones were selected and were subsequently transformed into Rosetta *E. coli* strains (Genlantis) for protein expression.

ILs. Commercially available ILs, listed in Table 1, were obtained from several vendors, Sigma-Aldrich, IoLiTec (Tuscaloosa, AL, USA), and Solvent Innovations. One IL, N-Butyl, N-methylpyrrolidinium dihydrogen phosphate, was synthesized as previously described [2]. Stock solutions of all ILs, except for ECOENG 500, were prepared as a 1 M solution in dH_2_O. Due to its high viscosity and MW, ECOENG 500 was prepared as a 0.5 M solution.

### 2.2. Methods

Protein expression and purification: Protein was expressed in 10 L culture. Typically, 1 mL of a frozen glycerol stock solution of the *E. coli* expressing the protein of interest was added to 1 L of LB broth containing carbenicillin and chloramphenicol. This was incubated with shaking overnight at 37 °C, after which the cells were collected by centrifugation, resuspended in a minimal amount of Terrific Broth (TB) media, and used to inoculate the large-scale fermentation. The fermentation vessel was a 20 L plastic carboy charged with 10 L of autoclaved TB media, 100 μg/mL carbenicillin (GoldBio, St. Louis, MO, USA, Cat.# C-103), 20 μg/mL chloramphenicol (GoldBio, St. Louis, MO, USA, Cat.# C-105), and 1 mL of antifoam (Sigma, Tuscaloosa, AL, USA, Cat.# A6457). The culture was aerated by two tygon tubing air lines that passed through holes in the carboy cap, with short pieces of stainless-steel tubing at the ends, closed off with a bolt and with multiple small holes drilled in them. The carboy is warmed by two high intensity lights placed ~12” from the carboy. After inoculation, the culture OD600nm was monitored and when it reached 0.6 protein expression was induced by adding isopropyl-β-D-thiogalactoside (IPTG, GoldBio, St. Louis, MO, USA, Cat. # 12481C) to a final concentration of 0.5 mM, at which point the lights were turned off and expression allowed to proceed at room temperature. Between 16 and 18 h after induction cells were harvested by centrifugation (8000× *g*, 15 min) and cell pellets of ~20 gms were stored at −80 °C. IPPase proteins were purified by thawing the cell pellets in lysis buffer (0.025 M Tris-HCl, 0.5 M NaCl, 0.005 M Imidazole, pH 8.2), followed by lysis using sonication. The supernatant was recovered by centrifugation (15,000× *g*, 20 min), which was then applied to a 5 mL Ni++ affinity column (GoldBio, St. Louis, MO, USA, Cat.# H-320-100) equilibrated in lysis buffer. The column was washed with 10 column volumes of lysis buffer, then eluted with a 120 mL gradient from lysis buffer to 0.025 M Tris-HCl, 0.5 M NaCl, 0.4M Imidazole, pH 8.2. The protein peak was identified by SDS gel electrophoresis, concentrated by ultrafiltration using a 30 kDa membrane, and then passed down a S-200 column (1.5 × 75 cm) equilibrated in 0.025 M Na-Hepes, 0.05 M NaCl, pH 7.5, at a flow rate of 1 mL/minute. The peak fractions were identified by SDS gel electrophoresis and concentrated by centrifugal ultrafiltration to a concentration >15 mg/mL. The 6XHis tags were not removed prior to crystallization trials.

Initial protein crystallization screening. All proteins were trace fluorescently labeled (TFL) with carboxyrhodamine (CR, Invitrogen, C-6157) as previously described [22,23]. Prior to IL optimization tests the proteins were subjected to two initial rounds of crystallization screening. The first round used the Hampton Research High Throughput screen (Hampton Research, Aliso Viejo, CA, USA, cat.# HR2-130), the JCSG+ screen (Molecular Dimensions, Maumee, OH, USA, cat.# MD1-40), the MCSG-3 screen (Anatrace, Maumee, OH, USA, cat.# 50-109-1514), and a 96-condition screen (Screen 4a) under development in-house to complement the above 3 screens [24]. Crystallization screening was carried out using Corning CrystalEX sitting drop plates (Hampton Research, Aliso Viejo, CA, USA, Cat.# HR8-140), with a reservoir consisting of 50 μL of precipitant solution and protein:precipitant drop ratios of 1:1, 2:1, and 4:1. All proteins were used at 12 mg/mL in the screening trials. The plates were incubated at ambient temperature, with periodic fluorescence imaging using a Crystal X2 plate imaging system [23]. After 6 weeks the screening plates were manually scored and the results subjected to Associated Experimental Design (AED) analysis [24]. A second round 96 condition screen, based on the AED analysis results for that protein, was formulated and the plate then set up using freshly prepared protein, again with periodic fluorescence imaging. After 6 weeks these results were scored and 12 conditions selected for subsequent IL optimization analysis. Overall about 2/3 of the selected conditions were those giving precipitated protein (scores of 0 or 1) or precipitated protein with bright spots (score of 4 [23]), with the balance being those giving crystals (scores of 8, 9), plates (score of 7), needles (score of 6), or urchins, spheroids and dendrites (score of 5).

IL optimization screening. Stock IL solutions are prepared at 1.0 M in dH_2_O. Aliquots of the stock solutions are dispensed into a deep well block for pipetting. Screening reservoir solutions were prepared by mixing stock IL solutions with precipitant. With 0.1 M IL as an example, using an eight-channel pipette 5 μL of IL solution is dispensed in the X direction in the reservoirs across the plate. Then, 45 μL of precipitant is dispensed in the Y direction down the plate, using a twelve-channel pipette. Thus, each row is constant in an IL to be tested and each column is constant in one of the 12 precipitant solutions. The solutions are mixed using a plate shaker. A total of 23 different ILs were tested, with the top row of the first plate for each IL concentration being a dH_2_O control. Three plates are used to test one IL concentration. IL concentrations of 0.2 and 0.4 M in precipitant are prepared by making the appropriate adjustments to the pipetted IL and precipitant volume ratios. This results in a total of nine plates for each protein.

Trace fluorescent labeling and plate imaging. All proteins were trace fluorescently labeled as previously described [23]. Crystallization progress was followed by periodic imaging of the screening plates using a Crystal X2 fluorescent imager (iXpressGenes Inc., Huntsville, AL, USA) At the end of the 6 to 8 week incubation period the results of each well were scored by hand, using white light microscopy. These scores were then adjusted as necessary by reference to the corresponding fluorescent images for the final score [23].

## 3. Results

The ILs used, listed in Table 1, were selected to give a representative range of anions and some of the most common cations that were commercially available at the time. One selection criteria was that they be soluble in dH_2_O. All ILs except ECOENG 500 were used as 1 M stock solutions in dH_2_O. Due to its high MW, ECOENG 500 could only be prepared as a 0.5 M solution.

**Figure 1.**
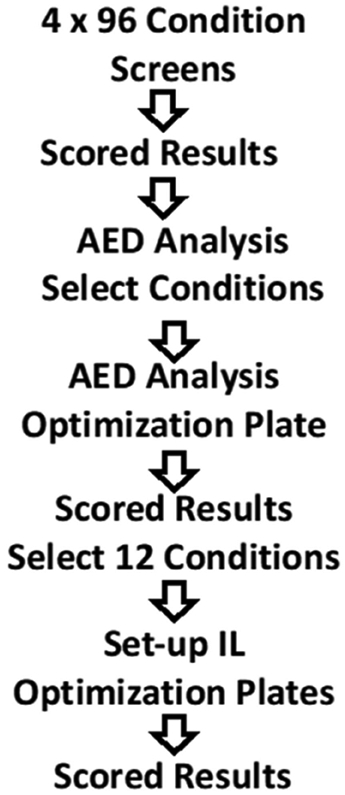
The flow diagram for the crystallization screening scheme.

**Figure 2.**
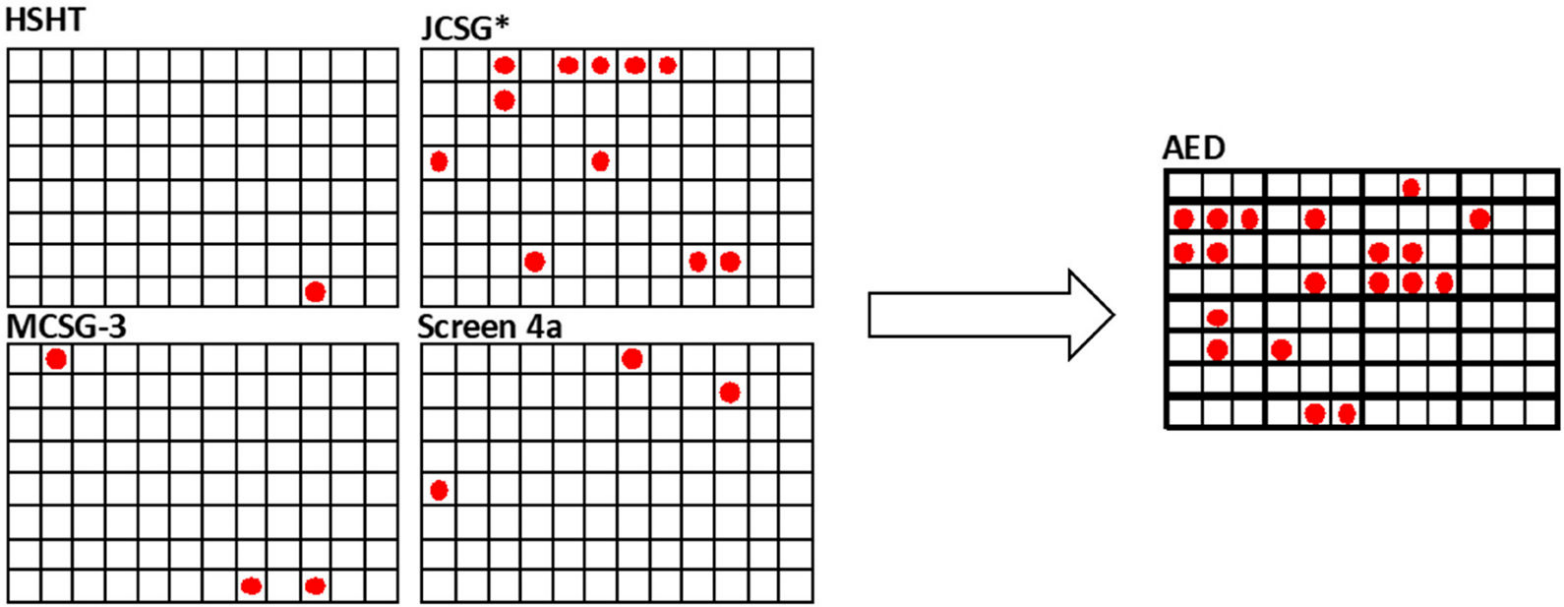
4 × 96 condition screening results to results after AED analysis for *Kp*IPPase. The right side shows where crystals were obtained for the indicated screens (red dots) using the four screens initially used with the protein. After AED analysis of the results, the new screen gave the results shown on the right side. The AED screen is 32 conditions in groups of three that vary in the precipitant concentration.

The crystallization plate screening flow is shown in Figure 1. Initial screening, using four 96 condition screens, was carried out. All wells of the crystallization plates are scored using a scale from 0 to 9, with a score of −1 reflecting a null, likely mis-pipetted, drop. This subjective scoring scheme reflects the experimenters perceived desirability of the possible outcomes, based on the empirical scoring scheme put forth by Brodersen et al. [25]. The crystalline nature of the outcomes is determined by the use of TFL, high fluorescence intensity being associated with more dense packing of the fluorescent probes and higher packing density being a function of their crystalline state. After 6 weeks, the scores for each condition were first determined by visual observation. These scores were then adjusted as needed by referring to the final fluorescent observation, which was used to eliminate salt crystals from the scores or to discover crystalline or visually indeterminant highly fluorescent objects, “bright spots” [23]. The scored screening conditions were then combined and subjected to AED analysis [24]. The software outputs a listing of novel conditions that are likely to result in crystallization based upon analysis of the input scored data as well as a ranked listing of the solution components associated with the highest scores. The 32 top conditions from the AED analysis are used to formulate a 96-condition screen, using three different precipitant concentrations, typically 1×, 0.66×, and 0.5×, for each condition. After setting up, these plates are incubated for 6 weeks, with periodic imaging as above, before they are scored. The goal of the process to this point is to identify new and robust crystallization conditions, which are taken to be those where crystals are obtained at all three precipitant concentrations. A common occurrence was that more crystals were obtained in the single AED-based optimization screen than were obtained in all four of the original screens, as shown in Figure 2 for the protein KpIPPase. The AED-based conditions are all cocktail formulations that are not present in the original screens.

The AED optimization results indicated that many of the derived conditions were indeed crystallization conditions. However, as shown in Figure 2, crystals were not always obtained. Despite being based upon the computed optimal crystallization conditions, many of the outcomes were precipitates. To test if these might also be crystallization conditions, a selection of these AED-derived conditions was used as the starting point for subsequent IL-based optimization screening experiments. Conditions that gave 3D crystals (score of 8, 9), 2D plates (score of 7), needles (score of 6), non-faceted crystalline precipitate (score of 5), bright spots (score of 4), and precipitated protein (scores of 0 and 1 for heavy and light precipitation, respectively) were selected for subsequent IL optimization trials. Scores of 2 and 3 denoted clear solutions and phase separations, respectively.

**Figure 3.**
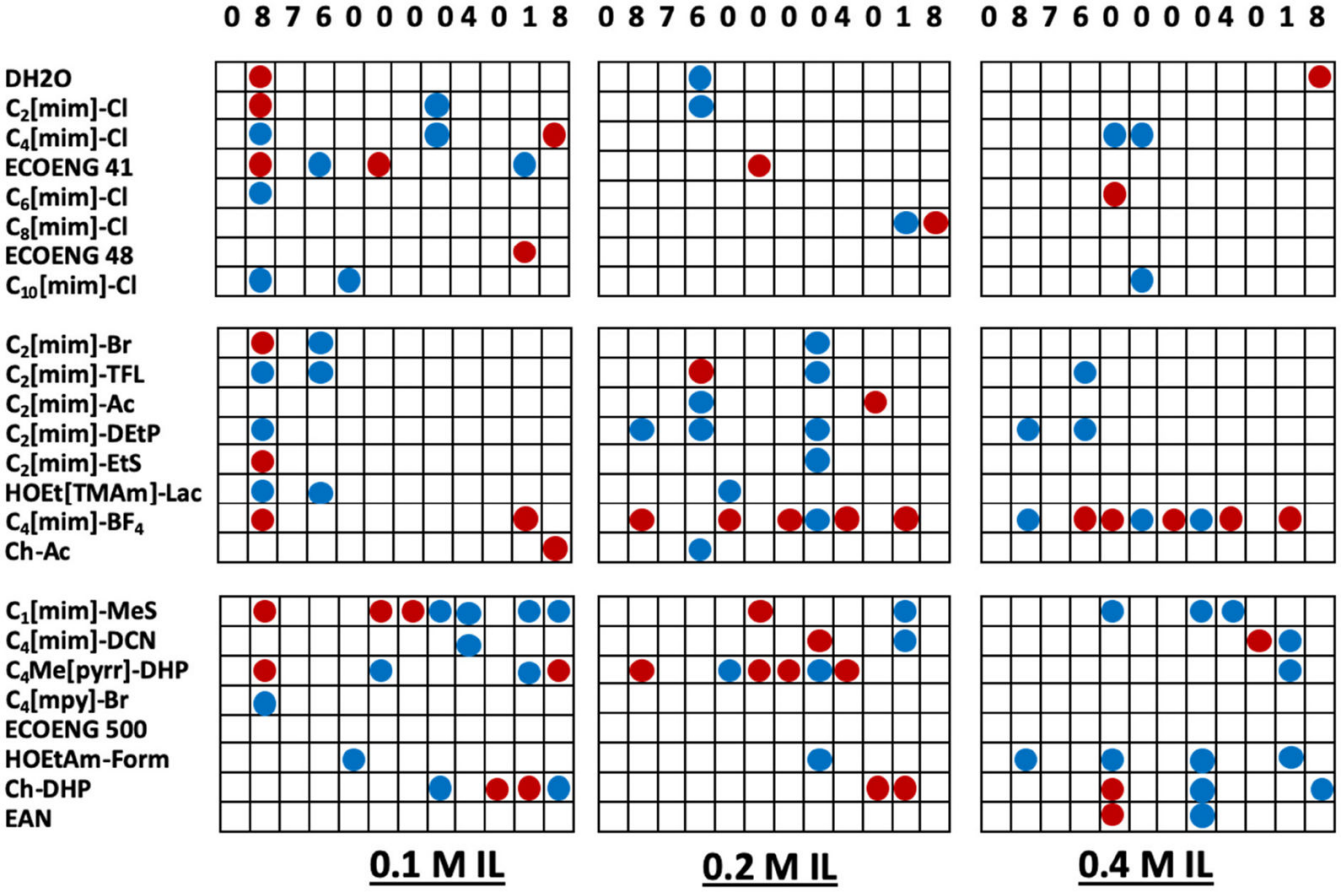
IL optimization of 12 of the KpIPPase AED optimization conditions. The ILs are listed on the left and the AED optimization condition scores are at the top of each block of conditions. The IL concentration is given under each block. Red dots are where 2D or 3D crystals were obtained from precipitation conditions. Blue dots are where non-faceted crystals (needles, urchins, spheroids, etc.) were obtained.

**Figure 4.**
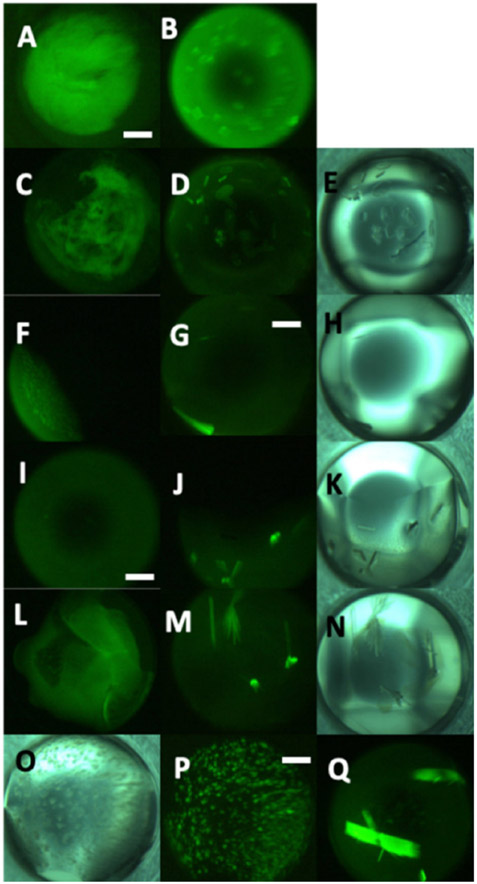
Examples of AED precipitation converted to crystallization conditions in the presence of IL Legend: (**A**)—*Cj*IPPase AED precipitate, (**B**)—Crystallization of A with 0.1 M HOEt[TMAm]-Lac, (**C**)—*Hi*IPPase AED precipitate, (**D**,**E**)—fluorescence and white light images of (**C**) with 0.4 M ChAc, (**F**)—hTFN AED precipitate, (**G**,**H**)—fluorescence and white light images of F with 0.1 M C_2_ [mim]-TFL, (**I**)—*Kp*IPPase AED precipitate, (**J**,**K**)—fluorescence and white light images of I with 0.1 M C_4_[mim]-BF_4_, note the salt crystal to the lower left of center of (**K**) that does not appear in (**J**), (**L**)—RrP42 AED precipitate, (**M**,**N**)—fluorescence and white light images of (**L**) with 0.2 M HOEtAm-Form, (**O**,**P**)—white light and fluorescence images of *Spn*IPPase non-faceted crystals (granular precipitate), and (**Q**)—fluorescence image of (**O**,**P**) in the presence of ECOENG 41.

IL optimizations were carried out on eight microbial IPPases, in addition to three non-IPPase proteins, with the ILs at 0.1, 0.2, and 0.4 M final concentrations in the precipitant solution. In preparing the IL-precipitant solutions the concentrations of the precipitant solution components are correspondingly reduced. This reduction is accounted for using an appropriate dH_2_O control for each IL concentration. Figure 3 shows outcomes for the IL optimization experiments for KpIPPase. These results are representative of those obtained for the other proteins. The numbers at the top of the columns show the scores of the parent AED screening condition from which that column of conditions was derived. Many of the ILs yielded crystals at a given IL concentration and precipitant condition, only to not yield crystals at a higher or lower IL concentration. Many of the dH_2_O control conditions for crystallization parent conditions did not give crystals, but did have hits at IL+ conditions. Additionally evident is the number of conditions where a precipitate was elevated to a non-faceted crystalline outcome in the presence of IL. In the absence of any crystals, these could be used for subsequent seeded crystallizations [26,27]. For all but one of the proteins, we have obtained crystals from conditions that only gave precipitant for the AED round of screening. Some examples are shown in Figure 4.

Trace fluorescent labeling (TFL) was necessary to interpreting the crystallization results. The ILs were often found to crystallize under the screening conditions with C_4_[mim]-BF_4_ being particularly notable in this regard, and TFL was used to distinguish protein from salt crystals. The salt crystals were apparently IL, and this was the only indication of any incompatibility with the screening cocktails. TFL removes this as a problem in interpreting the results. An example of this is shown in Figure 4, panels J and K. Additionally, by observing the fluorescence intensity one can use the TFL method to find hidden crystallization leads in plates that apparently had amorphous precipitate for the outcome or crystals mixed in the precipitate [23].

The AED conditions tested varied from protein to protein. For these experiments, only 12 precipitant solutions needed to be prepared in bulk for each protein, ensuring consistency in the solution conditions. A total of 12 conditions/protein × 11 proteins, or 132 conditions were tested. However, each condition was tested with 3 IL concentrations, giving 396 conditions. Using 23 ILs and a dH_2_O control, this gives 9504 experiments. For the 11 proteins, 261 (66%) of the 396 tested conditions were derived from AED precipitation outcomes, 42 (10.6%) were from AED conditions that gave non-faceted crystals, and 93 (23.5%) were from AED conditions that gave faceted crystals. Crystals were obtained for 95 (36.4%) of the 261 precipitation conditions with at least one IL, 18 (42.8%) of the 42 non-faceted conditions, and 53 (60%) of the 93 crystallization conditions. Increasing the IL concentration, with a concomitant decrease in the precipitation solution concentrations, affected the success rate. Over all the hits, for precipitate to crystals, 0.1 M IL was responsible for 39%, 0.2 M for 35.7%, and 0.4 M for 25.2% of the conditions yielding crystals. For improving non-faceted crystals to crystals, 0.1 M gave 47.8%, 0.2 M 32.6%, and 0.4 M 19.6% of the hits.

It is possible that the IL results are only valid for IPPases. While all the IPPases tested share the same catalytic function, there is considerable variation in their sequences. Table 2 shows the sequence identities calculated by Clustal Omega [28-30] and includes *E. coli* IPPase for reference. S. pyrogenes and S. pneumonia IPPases are type II Mn++-dependent activities, while the others are type I Mg++-dependent activities [15-18]. The IPPases also had a considerable variation in crystallization propensity and conditions. The three non-IPPase proteins represent a limited sample from which to draw conclusions, but the overall IL-derived success rate for these proteins was comparable to that for the IPPases.

**Figure 5.**
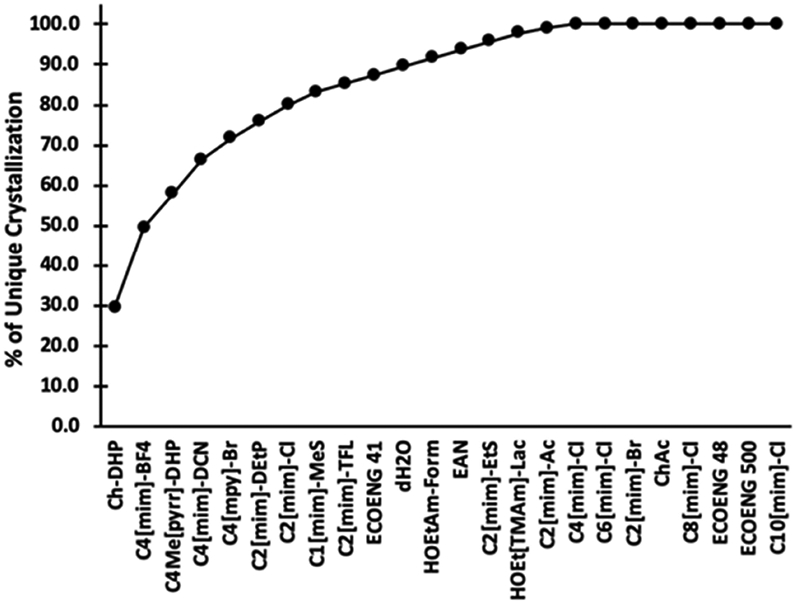
The incremental success rate for the tested ILs. The number of unique hits for each IL, those where a previous IL did not give crystals from precipitated protein conditions with the same precipitant solution at the same IL concentration, are determined and plotted as the incremental percentage success rate. Note that the ILs from C_8_[mim]-Cl to C_10_[mim]-Cl gave crystals, but that crystals had been obtained for those conditions by other ILs.

The crystallization data were analyzed to determine which ILs were most effective. Three levels of outcome are considered; first the conversion of apparent precipitate to 2D or 3D crystals, second the conversion of non-faceted crystals to 2D or 3D crystals, and third all crystallization outcomes, which is essentially the first two plus where crystals are obtained from known IL crystallization conditions. These results are summarized in Table 3. One finding is that simple dilution of a precipitant solution with dH_2_O has an appreciable effect on the outcomes obtained for the conversion of precipitation to crystallization conditions. These data serve as a starting point for subsequent selection and design of ILs for protein crystallization applications. The actual effectiveness for each IL was determined by first determining the overlap between ILs in Table 3, working down from the most effective to the least. Using the results for KpIPPase, Figure 3, as an example, the IL Ch-DHP gave crystals for 5 conditions, two at 0.1 and 0.2 M each, and 1 at 0.4 M. The second IL in Table 3, C_4_[mim]-BF_4_, resulted in crystals for 12 conditions (2 at 0.1 M, 5 at 0.2 M, and 5 at 0.4 M). However, one C_4_[mim]-BF_4_ condition each at 0.1, 0.2, and 0.4 M is at the same precipitate condition as for Ch-DHP at those IL concentrations. Thus, there are 5 crystallization conditions that would be obtained by Ch-DHP and 9 new conditions that would be obtained by C_4_[mim]-BF_4_, for a total of 14 conditions that would be had with just these two ILs. Going through Table 3 with this approach the 95 unique IL-derived crystallization conditions are ranked as shown in Figure 5. While Ch-DHP and C_4_[mim]-BF_4_ are still the top ranked ILs, the subsequent order of effectiveness changes from that shown in Table 3. While no one IL will be universally applicable, a useful functional set can be chosen such that the broadest range of utility is obtained. This analysis also indicates that those ILs that were least effective. Although they did give crystals, they did not add any new crystallization conditions. Figure 5 summarizes this analysis for all of the ILs tested, plus the dH_2_O control, in order of decreasing effectiveness. This results in a different ordering in terms of effectiveness in comparison to Table 3. By the eighth IL, C1[mim]-MeS, 83% of the new crystallization outcomes are obtained. From Table 3, all of the ILs yielded crystals for at least one of the tested conditions. However, from Figure 5, the ILs C_6_[mim]-Cl through C_10_[mim]-Cl did not add any new crystallization conditions compared to Ch-DHP through C_4_[mim]-Cl. This analysis is for crystallization only and does not take into consideration where the IL may have an effect on the diffraction data obtained (manuscript in preparation).

The data were broken down to see if the same trends in IL effectiveness as shown in Table 3 held for all classifications of precipitants. The classifications tested were diacids, salts (phosphate, sulfate, halides), low MW PEGs (to ≤2 K), intermediate MW PEGs (3 K to 8 K), high MW PEGs (>8 K), diols (MPD, butanediol, hexanediol), all PEG MMEs, PPG 400, and PVP. Approximately the same trends as shown in Figure 5 were observed for each of these classifications, with either Ch-DHP or C_4_[mim]-BF_4_ as the leading IL. For several of the precipitant classifications (diols, HMW PEGs), there were insufficient instances from which to draw conclusions. The precipitant classification range with the most data was for PEG 3 K to 8 K, the results for which are shown in Table 4. The data are sorted by the conversion of precipitated protein to crystals. The differences between this ordering and that shown in Table 3 and Figure 5 suggest that there may be a precipitant-based dependence upon the effectiveness of a given IL. If the data included in Table 4 ranking are expanded to all of the PEGs, then five each of the top and bottom eight ILs in Table 4 are present in the top and bottom eight of the expanded listing, respectively. The same ILs are in the same order for the top and bottom three positions, respectively, and the differences are in the intermediate ILs.

**Figure 6.**
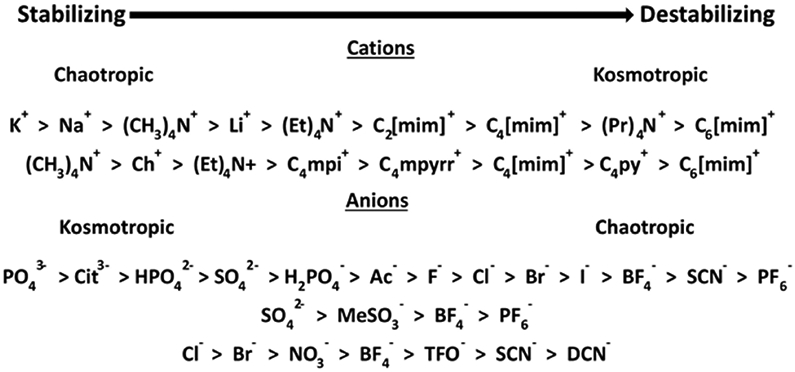
Extended Hofmeister series including some common IL cations [31,33] and anions [32-35]. Abbreviations: mpyrr, methylpyrrolidinium; mpi, methylpiperidinium; TFO, trifluoromethanesulfonate; py, pyridinium; mim, methylimidazolium; ch, choline.

ILs are salts, and as such can be placed in the Hofmeister series. It is reasonable to ask if the position of a given IL in this series determines its effectiveness in promoting crystallization. Figure 6 gives a composite Hofmeister series for anions and cations, including those used for ILs, derived from the literature [31-35]. The top two ILs in Table 1, Ch-DHP and C_4_[mim]-BF_4_, are in opposite regions of the series. In fact, the top four ILs alternate back and forth between pairs of stabilizing and destabilizing ions. This is illustrated in Table 5, where the hits for a given IL that overlap with one of the other top four ILs are determined. The destabilizing ILs are shadowed in gray, and we see that there is more overlap of the stabilizing with the destabilizing ILs than there is for the other stabilizing ILs and vice versa. The effectiveness of the separate IL anions and cations can be inferred from these results. For example, for the Cl anion, the alkyl methylimidazolium cation order does follow the Hofmeister series and is C_2_ > C_4_ > C_6_ > C_8_ > C_10_ for obtaining crystals from precipitate.

Only one protein, SpyIPPase, did not have any precipitation conditions converted to faceted crystals for any of the ILs. The occurrence of IL+ crystalline outcomes was often IL concentration dependent. A common outcome in the precipitation condition derived tests was the presence of non-faceted crystals in the IL+ outcomes. In many cases, there would not be any success for any IL with a given precipitate at one concentration, then one or more ILs would result in crystals at another, either at higher or lower concentrations. There were also many conditions that did not give crystals, faceted or not, at any IL+ concentration. This may be due to the IL not being suitable or, more likely, that these were not valid crystallization conditions.

## 4. Conclusions

We find that ILs can be used to salvage crystallization conditions from amorphous precipitation outcomes. The major loss of targets when going from gene to structure is in the protein crystallization step. The most common outcome in screening trials is a plate of precipitated protein, which gives little to no feedback to guide subsequent trials. ILs provide a simple and convenient approach to potentially salvaging crystallization conditions from this outcome without having to resort to chemical or genomic modification. Other losses occur when proteins are expressed as inclusion bodies, and when the crystals that are obtained do not diffract to sufficiently high resolution. As we show above, off-the-shelf ILs can be used to obtain crystals from apparently otherwise failed outcomes (precipitate). Other published data indicate that ILs are useful for refolding protein [8,9], and to obtain better diffracting crystals from sub-optimal starting outcomes [3]. Thus, ILs have applications for all phases of the protein crystallization process. The problem is, which to use?

This work focuses on the use of ILs as additives for protein crystallization. There are three levels of outcome improvement, the first two of which are considered here. First, obtaining crystals from otherwise non-productive precipitation conditions. The nature of the precipitation conditions that benefitted the most, whether denaturing or not, remains to be determined, and likely hinges on whether a given IL improves the protein’s stability or solubility, which effect may be protein and/or precipitate specific. Second is upgrading non-faceted crystallization outcomes, aka needles, spheroids, urchins, granular precipitate, etc., to faceted 2D or 3D crystals. Third is improving diffraction data quality from existing crystallization conditions. While several ILs may result in crystals for a given precipitation condition, the effects on the diffraction data quality may be IL dependent (manuscript in preparation). Diffraction data will also show if the ILs are binding to the protein.

The major advantage from these experiments is obtaining crystals from outcomes that would otherwise have been considered to be failures. The majority (66%) of the tested conditions were a precipitate in the AED optimization plates. Beyond that the non-productive tested conditions may not actually be crystallization conditions, a limiting factor in the IL success rate may be that the precipitates were due to denatured protein, that the ILs were only positively affecting soluble precipitation conditions. Most of the tested precipitation conditions employed PEGs, which have been shown to destabilize protein structure [36-39]. In some cases, the ILs may be providing a stabilizing effect that offsets the destabilizing effect of the PEG. There is great utility in recovering crystals from precipitation conditions. As no crystals is the likely default outcome from screening experiments, this presents an alternative path forward without having to return to the genome or employ other protein modification techniques.

Given the number of plates that were set up for these studies, the routine use of ILs as optimization additives using this approach is not practical. However, the obtained results indicate which of the 23 ILs employed are the most effective. Just two of the ILs, Ch-DHP and C_4_[mim]-BF_4_, account for ~50% of the unique crystallizations in the optimization plates while the top eight ILs bring this to ~83%. A more limited set for crystallization use can be derived from these results. What is not known is what makes a given IL more or less effective, and whether it links to the protein and/or precipitant solution characteristics. Alternatively, they may be stabilizing the protein, such that the desolubilization occurs as a crystalline rather than a denatured protein precipitate [12,14].

It is apparent that the IL position on the Hofmeister series, or even the position of either of the constituent anions or cations, does not *a priori* determine effectiveness in protein crystallization. Optimal protein stability is typically from chaotropic cations and kosmotropic anions [40] (Yang, 2009). Protease stability is reduced by the chaotropic anion BF_4_- and kosmotropic cation C_4_[mim]^+^ [34]. However, the thermal stability of horseradish peroxidase was found to be improved by C_4_[mim]-BF_4_ [33]. Ch-DHP better fits the chaotropicity/kosmotropicity paradigm, with a strongly chaotropic cation and a kosmotropic anion. Ch-DHP with 20% added H_2_O was found to stabilize cytochrome c activity at room temperature for 18 months [41]. Ch-DHP stabilized human serum albumin fatty acid binding while C_4_[mim]-BF_4_ apparently promoted unfolding [42]. Unfolding of myoglobin by guanidinium HCl was facilitated by C_4_[mim]-BF_4_ at 0.15 M, while it was unchanged in the presence of C_2_[mim]-Ac [43]. The thermal stability of ribonuclease A was enhanced by Ch-DHP while C_2_[mim]-DCN was a strong denaturant [44]. The IL effects may come from specific IL–protein interactions or they may derive from interactions with the other solution components. However, from the limited data available, we cannot determine if specific ILs work best with specific types or classes of solution component.

Just prior to this manuscripts submission, Shaposhnikova et al. [45] published results showing that Rhodococcus rhodochrous haloalkane dehalogenase structure is stabilized by bound ILs. The two ILs, 2-hydroxyethylammonium acetate (C_2_OH[mim]-Ac) and 1-butylmethylimidazolium methyl sulfate (C_4_[mim]-MeS), were found in the active site region by X-ray crystallography. Subsequent molecular dynamics (MD) studies showed that increasing concentrations of the ILs both strengthened and stabilized the secondary and tertiary structures of the protein. Further, the C_2_OH[mim] cation was shown by MD studies to interact with the proteins hydrophobic surface. The presence of the ILs in the solution also led to an indirect stabilizing effect by disrupting protein–water contacts, further contributing to structural stability. In contrast to the work presented in this above, the ILs were not present in the crystallizing step but introduced to the protein by soaking the crystals in an IL+ solution.

The goal of this project was to first test if the AED-derived crystallization conditions had “missed” crystallization conditions. The IL+ results clearly show that many of these AED-derived conditions were in fact crystallization conditions. Secondly, we sought to survey which ILs might be most useful for optimization of crystallization conditions. Those ILs at the top of the list were the most effective at yielding crystals, but given the range of possible IL structures they may not be the best. From Figure 5 the most prevalent cations are methyl, ethyl, and butyl based. Five of them have a methylimidazolium cation, and two of them have a dihydrogen phosphate anion. A third phosphate moiety appears as a diethylphosphate. Of particular interest is that four of the cations have a butyl group.

This work assesses the effectiveness of different ILs as aids in the crystallization of proteins. The data demonstrate that the ILs varied considerably in their effectiveness. The major role that was investigated was in recovering crystals from outcomes that gave amorphous precipitate in “standard” screening tests, which presents an attractive means of target recovery at the crystallization stage for structural studies. While the selected conditions can be considered to be biased as having a likely high likelihood for crystallization, still of the 261 precipitation conditions tested 36.4% of them were converted to crystals by one or more ILs. The mechanism by which the ILs are affecting the crystallization is not readily apparent. A clear pattern is also not apparent in the Hofmeister series. However, the data do indicate potential directions for further explorations into the optimizing of IL structure for use in protein crystallization.

## Figures and Tables

**Table 1.** ILs used in this study.

IL	Abbreviation	Source
1-Ethyl-3-methylimidazolium chloride	C_2_[mim]-Cl	Fluka
1-Butyl-3-methylimidazolium chloride	C_4_[mim]-Cl	Sigma
1-Hexyl-3-methylimidazolium chloride	C_6_[mim]-Cl	Solvent Innovations
1-Octyl-3-methylimidazolium chloride	C_8_[mim]-Cl	Iolitec
1-Decyl-3-methylimidazolium chloride	C_10_[mim]-Cl	Sigma
1-Butyl-3-methylimidazolium diethyleneglycol monomethylether sulfate	ECOENG 41	Solvent Innovations
1-Octyl-3-methylimidazolium diethyleneglycol monomethylether sulfate	ECOENG 48	Solvent Innovations
1-Methyl-3-methyl-midazolium methyl sulfate	C_1_[mim]-MeS	Solvent Innovations
1-Ethyl-3-methylimidazolium diethyl phosphate	C_2_[mim]-DEtP	Iolitec
1-Ethyl-3-methylimidazolium triflate	C_2_[mim]-TFL	Iolitec
1-Ethyl-3-methylimidazolium acetate	C_2_[mim]-Ac	Iolitec
1-Ethyl-3-methylimidazolium ethylsulfate	C_2_[mim]-EtS	Iolitec
1-Ethyl-3-methylimidazolium bromide	C_2_[mim]-Br	Fluka
1-Butyl-3-methylimidazolium tetrafluoroborate	C_4_[mim]-BF_4_	Sigma
1-Butyl-3-methylimidazolium dicyanamide	C_4_[mim]-DCN	Iolitec
Choline dihydrogen phosphate	Ch-DHP	Iolitec
N-Butyl, N-methylpyrrolidinium dihydrogen phosphate	C_4_Me[pyrr]-DHP	[2]
N-Methyl-N-butyl-pyridinium bromide	C_4_[mpy]-Br	Solvent Innovations
Ethyl ammonium nitrate	EAN	Iolitec
2-Hydroxyethyl-trimethylammonium L-(+)-lactate	HOEt[TMAm]-Lac	Sigma
Choline acetate	Ch-Ac	Sigma
2-Hydroxyethylammonium formate	HOEtAm-Form	Iolitec
Cocosalkyl pentaethoxy methylammonium Methylsulfate	ECOENG 500	Solvent Innovations

**Table 2.** Sequence identities of the IPPases.

	E. coli	S. typhimurium	F. tularensis	C. jejuni	K. pneumoniae	H. influenzae	A. baumannii	S. pyrogenes	S. pneumoniae
*E. coli*	100.00	94.32	63.01	51.16	42.86	33.33	67.43	18.67	19.28
*S. typhimurium*	94.32	100.00	61.27	50.00	41.71	33.33	65.14	18.67	19.28
*F. tularensis*	63.01	61.27	100.00	52.05	42.44	35.09	56.07	16.46	17.68
*C. jejuni*	51.16	50.00	52.05	100.00	40.70	31.58	49.42	21.47	19.63
*K. pneumoniae*	42.86	41.71	42.44	40.70	100.00	32.00	40.23	14.12	16.47
*H. influenzae*	33.33	33.33	35.09	31.58	32.00	100.00	31.79	16.27	15.06
*A. baumannii*	67.43	65.14	56.07	49.42	40.23	31.79	100.00	21.21	21.21
*S. pyrogenes*	18.67	18.67	16.46	21.47	14.12	16.27	21.21	100.00	82.64
*S. pneumoniae*	19.28	19.28	17.68	19.63	16.47	15.06	21.21	82.64	100.00

**Table 3.** Effectiveness of the ILs tested.

IL	Pcpt→Xtl	Non-Faceted→Xtl	All
Ch-DHP	28	5	49
C_4_[mim]-BF_4_	22	4	43
C_4_Me[pyrr]-DHP	14	1	26
C_4_[mim]-DCN	11	0	22
C_1_[mim]-MeS	11	0	18
C_2_[mim]-DEtP	10	4	28
C_2_[mim]-TFL	9	3	25
C_4_[mpy]-Br	9	2	21
ECOENG 41	9	1	14
C_2_[mim]-Cl	9	0	25
C_2_[mim]-Ac	8	4	19
dH_2_O	8	3	29
HOEtAm-Form	8	3	20
EAN	8	2	23
C_2_[mim]-EtS	8	1	24
HOEt[TMAm]-Lac	6	4	17
C_4_[mim]-Cl	6	3	30
C_6_[mim]-Cl	6	2	14
C_2_[mim]-Br	5	2	23
ChAc	5	1	20
C_8_[mim]-Cl	5	1	10
ECOENG 48	3	0	5
ECOENG 500	2	0	8
C_10_[mim]-Cl	0	0	6

**Table 4.** IL optimizations for PEGs 3 K to 8 K.

IL	Xtl→Xtl	5,6→Xtl	0–4→Xtl
C_4_[mim]-BF_4_	6	1	13
Ch-DHP	3	6	8
C_1_[mim]-MeS	2	0	8
C_4_Me[pyrr]-DHP	2	0	7
C_2_[mim]-TFL	4	2	5
C_2_[mim]-Ac	3	2	5
C_2_[mim]-DEtP	4	2	5
ECOENG 41	0	0	5
HOEt[TMAm]-Lac	3	3	4
HOEtAm-Form	2	2	4
ChAc	5	1	4
C_2_[mim]-Cl	7	0	4
C_4_[mim]-DCN	5	0	4
C_6_[mim]-Cl	2	2	3
EAN	3	2	3
C_2_[mim]-Br	5	1	3
C_2_[mim]-EtS	7	1	3
C_4_[mpy]-Br	2	0	3
ECOENG 500	1	0	3
dH_2_O	5	2	2
C_4_[mim]-Cl	7	1	2
C_8_[mim]-Cl	1	1	2
ECOENG 48	0	0	1
C_10_[mim]-Cl	3	0	0

**Table 5.** Overlap in crystallization hits for the top four ILs.

	Ch-DHP	C_4_[mim]-BF_4_	C_4_Me[pyrr]-DHP	C_4_[mim]-DCN
Total Instances	49	42	26	22
Ch-DHP	100%	21% (9)	15% (4)	23% (5)
C_4_[mim]-BF_4_		100%	27% (7)	15% (3)
C_4_Me[pyrr]-DHP			100%	5% (1)
C_4_[mim]-DCN				100%

The % is for the number of the total number of hits of that IL that overlap with the intersecting IL.

## Data Availability

The data has not been publicly archived.
